# The profile and frequency of known risk factors or comorbidities for deep vein thrombosis in an urban district hospital in KwaZulu-Natal

**DOI:** 10.4102/sajhivmed.v17i1.425

**Published:** 2016-05-13

**Authors:** Damilola Awolesi, Mergan Naidoo, Mohammed H. Cassimjee

**Affiliations:** 1 Department of Family Medicine, University of KwaZulu-Natal, South Africa

## Abstract

**Background:**

Although deep vein thrombosis (DVT) is a preventable disease, it increases the morbidity and mortality in hospitalised, patients, resulting in considerable economic health impact. The identification and primary prevention of risk factors using risk assessment and stratification with subsequent anti-thrombotic prophylaxis in moderate- to severe-risk categories is the most rational means of reducing morbidity and mortality.

**Aim and setting:**

The aim of the study was to describe the profile and frequency of known risk factors or comorbidities of hospitalised medical patients with ultrasound-diagnosed DVT in an urban district hospital in KwaZulu-Natal.

**Methods:**

A retrospective review of clinical notes of all medical patients (age ≥ 13 years) admitted to the hospital with ultrasound-diagnosed DVT between July and December 2013.

**Results:**

The median age was 40 years (interquartile range 32–60 years) and female preponderance was 72.84%. HIV and tuberculosis emerged as the prevalent risk factors, accounting for 51.85% and 35.80%, respectively. Other risk factors observed were recent hospitalisation (34.57%), smoking (25.93%), previous DVT (19.75%) and congestive cardiac failure (18.52%).

**Conclusion:**

DVT in our study occurred predominantly in young female patients unlike previous studies where patients were generally older. Furthermore, HIV and tuberculosis were the two most common known risk factors or comorbidities observed. Clinicians should have a heightened awareness of venous thromboembolism in patients with either condition or where both conditions occur together and appropriate thromboprophylaxis should be administered.

## Introduction

Deep vein thrombosis (DVT), with an average annual incidence of 48 per 100 000 persons, accounts for more than half of venous thromboembolic events in the United States and 52 per 100 000 persons in Australia.^1,2^ In India, an overall incidence of confirmed DVT has been shown to be 17.46 per 100 000 patients with 64% occurring in non-surgical non-trauma patients.^3^ Over 200 000 South Africans suffer from DVT each year, and because most DVT is occult, the true incidence is unknown.^4^ According to statistics South Africa, thromboembolic disease is responsible for 20 000 deaths per annum in South Africa.^5^ However, there are currently no studies describing the prevalence of DVT in KwaZulu-Natal. The economic impact of venous thromboembolism (VTE) is enormous, as shown in a systematic review conducted in high-income countries (HIC) which showed that the economic impact on the healthcare system was because of the initial VTE diagnosis and treatment as well as the costs involved in managing recurrent hospitalisations.^6^

Although DVT is a preventable disease in hospitalised patients, morbidity and mortality associated with DVT is still high in these patients.^7^ There are several well-defined studies documenting risk in medical patients and the impact of thromboprophylaxis.^8^ In adults, several clinical conditions have been documented to predispose to VTE, and these include increasing age, stroke or paralysis, previous VTE, congestive heart failure, acute infection, pregnancy or puerperium, cancer and its treatment, prolonged immobility, dehydration, hormonal treatment, varicose veins, long-distance air travel, acute inflammatory bowel disease, rheumatological disease and nephrotic syndrome.^9^ Oral contraceptive pills, especially those that contain third-generation progestins, also increase the risk of VTE.^10^ There is a strong association between recent respiratory infection and VTE as described by Clayton et al.^11^ There are many well-described anomalies predisposing HIV-infected patients to developing VTE.^11^ Genetic factors such as primary coagulation abnormalities (e.g. deficiency of proteins C and S, anti-thrombin III, factor V Leiden, prothrombin 20210A) have also been documented to increase the risk of VTE.^12^

Recent reports suggesting increased risk of DVT amongst patients with HIV and tuberculosis (TB) point to the increasing role of these infections in the development of DVT. Given the high burden of HIV in South Africa, it is not surprising that many of the medical patients admitted with DVT are infected with HIV. Many researchers have documented the positive correlation between thrombosis and HIV infection because HIV predisposes to a hypercoagulable state, particularly those with severe immunosuppression (CD4 < 200 cells/mL).^13,14^ A study conducted in 2009 at Nelson Mandela Hospital, Mthatha, showed that the prevalence of DVT was 12.5% amongst HIV-positive patients admitted to the medical wards.^15^

Identification of suspected DVT cases is difficult and sometimes these are missed.^16^ However, administering prophylaxis to all patients admitted to hospital is not cost-effective, especially in low- and middle-income countries.^17^ The high economic burden and increased morbidity and mortality associated with VTE make it important that primary prevention of DVT with risk assessment or stratification and subsequent anti-thrombotic prophylaxis in the moderate- to severe-risk category of patients is the most rational means of managing such patients.

The Padau prediction score, which assesses the risk of VTE and identifies potential high-risk hospitalised medical patients for appropriate thromboprophylaxis, makes use of clinical components to arrive at this scores.^18^ A score of < 4 is categorised as low risk whilst a score of ≥ 4 is regarded as high risk.^18^ Likewise, the Wells scoring system, which is a clinical prediction rule for estimating pretest probability of DVT and further subsequent diagnostic testing developed in 1997 by Wells et al.,^19^ has nine clinical components with a possible score range of -2–8, and this was risk stratified into three groups, namely, high risk (≥ 3 points), intermediate risk (1–2 points) and low risk (< 1 point). It was later modified in 2003 with the addition of a further component (previously documented DVT) which was observed to increase the risk of DVT, giving a possible score range of -2–9.^20^ This version reduced the risk categories into two groups, namely, likely (≥ 2 points) and unlikely (< 2 points), based on the symptoms, signs and risk factors. With the emergence of HIV and its well-documented predisposition for coagulopathy, HIV is yet to be considered as an additional risk factor in any tool aimed at assessing the risk category for thromboprophylaxis administration. Tables 1 and 2 show the modified Wells and Padau prediction scoring systems, respectively, with the assigned points for each clinical feature or component. A definitive diagnosis is made by Doppler ultrasound scan for patients stratified as likely, and the most reliable indicator of the presence of a thrombus within a vein is direct visualisation of intraluminal thrombus or non-compressibility of the vein during ultrasound.^21^ These operator-dependent techniques are highly accurate in the diagnosis of DVT with a weighted mean sensitivity and specificity of 97% and 94%, respectively, for proximal DVT.^22^ Use of clinical signs and symptoms alone for diagnosis of DVT cannot be relied upon because they do not reliably predict which patients have DVT.^16^ The sensitivity of clinical signs and symptoms alone ranges from 60% to 96% and the specificity from 20% to 72%; therefore, it is not recommended without objective non-invasive diagnosis.^16^

**TABLE 1:** The modified Wells scoring chart.

Clinical feature	Points
Active cancer (treatment ongoing, within 6 months, or palliative)	1
Paralysis, paresis or recent plaster immobilisation of the lower extremities	1
Recently bedridden for 3 days or more or major surgery within 12 weeks requiring general or regional anaesthesia	1
Localised tenderness along the distribution of the deep venous system	1
Entire leg swollen	1
Calf swelling at least 3 cm larger than asymptomatic side	1
Pitting oedema confined to the symptomatic leg	1
Collateral superficial veins (non-varicose)	1
Previously documented DVT	1
An alternative diagnosis is at least as likely as DVT	-2
**Clinical probability–simplified score**	-
DVT *likely*	2 points or more
DVT *unlikely*	1 point or less

*Source*: Wells et al.^20^

DVT, deep vein thrombosis.

**TABLE 2:** The Padau prediction scoring chart.

Risk factors or clinical features	Points
Active cancer	3
Previous venous thromboembolism	3
Reduced mobility	3
Thrombophilia	3
Recent trauma or surgery (less than 1 month)	2
Advanced age (> 70 years)	1
Heart or respiratory failure	1
Acute myocardial infarction or stroke	1
Acute infection and/or rheumatic disease	1
Obesity (BMI ≥ 30 kg/m^2^)	1
Ongoing hormonal treatment	1

*Source*: Barbar et al.^18^

**TABLE 3:** Demographic and clinical profile of the patients.

Variable	Male	Female
Number of patients (%)	22 (27.16)	59 (72.84)
Median age in years (interquartile range)	39 (32.5–61.5)	41 (32.5–58.5)
HIV-positive (%)	11 (13.58)	31 (38.27)
Tuberculosis (%)	9 (11.11)	20 (24.69)
Swelling (%)	20 (24.69)	56 (69.14)
Pain (%)	19 (23.46)	55 (67.90)
Fever (%)	3 (3.70)	13 (16.05)
Others (%)	2 (2.47)	7 (8.64)

Given the burden of HIV and TB amongst the patients seen in our study setting, we were interested in exploring the possible impact of these infections on the profile of patients presenting with DVT in our hospital. Therefore, the aim of the study was to describe the profile and frequency of risk factors of medical patients admitted with non-invasive ultrasound-diagnosed DVT.

## Research method and design

This was a retrospective descriptive study done by reviewing clinical notes at an urban district hospital in KwaZulu-Natal. The study topic was selected based on the researcher’s observation of an increased number of medical patients being admitted with DVT. The clinical records of all medical patients aged ≥ 13 years who were admitted to the medical wards with ultrasound-diagnosed DVT between 1 July and 31 December 2013 were retrospectively reviewed and relevant data were extracted using a pre-defined data extraction tool.^15^ Patients’ records were identified using the ultrasound unit register and the hospital admission or discharge register. Further review of clinical records and laboratory data (accessed from the National Health Laboratory Service database) was done in order to extract relevant parameters for the study. The data extraction tool used was developed from a validated tool used in a previous study.^15^ Data were captured onto Microsoft excel spreadsheet and imported into Stata version 13 for analysis. Frequency and proportional tables, mean, median, and standard deviation were generated to describe the findings.

## Ethical consideration

Ethical approval was obtained from University of KwaZulu-Natal biomedical research ethics committee (Ref.BE214/14) as well as KwaZulu-Natal Department of Health and the management of the hospital where the study was conducted.

## Results

From the hospital records, a total of 118 patients with suspected DVT based on their clinical history, signs and symptoms were sent for ultrasound scans during the study period. Eighty one (68.64%) of these patients had confirmed DVT, whilst 37 (31.36%) had negative ultrasound scan results. Of the 81 who had ultrasound-confirmed DVT, 52 (64.2%) presented at the emergency unit with signs and symptoms, whilst 29 (35.8%) developed DVT in the ward during admission for other medical conditions. The age distribution of those with confirmed DVT ranged from 19 to 80 years, with median age of 40 years (interquartile range [IQR] 32–60 years). Amongst those with confirmed DVT, 22 were male patients (27.16%), whilst 59 were female patients (72.84%).

The study showed that 78 patients (96.30%) had DVT of the lower extremity. The remaining three patients (3.7%) had upper extremity DVT: two were in the neck (internal jugular vein) and one in the right subclavian vein.

The most common clinical presentation found in the study sample was swelling and pain, accounting for 76/81 (93.83%) and 74/81 (91.36%), respectively. Fever was seen in 16 patients (16/81, 19.75%), whilst other presentations like shortness of breath, cold limb and skin changes were recorded in only 9 patients (9/81, 11.11%). Table 3 shows the demographic and clinical profile of the patients.

The most common risk factors identified in this study were HIV (*n* = 42) and TB (*n* = 29), whilst recent admission (*n* = 28), smoking (*n* = 21), previous DVT (*n* = 16) and congestive cardiac failure (*n* = 15) were other risk factors seen. Risk factors like myocardial infarction, pregnancy, recent surgery, immobility (unable to perform activities of daily living for ≥ 3 days), oral contraceptive pills (OCP) and hormone replacement therapy (HRT) accounted for a negligible proportion. Table 4 depicts the observed risk factors.

**TABLE 4:** Observed risk factors.

Risk factor	Frequency	%
Myocardial infarction	3	3.70
Malignancy	4	4.94
Previous deep vein thrombosis	16	19.75
Congestive cardiac failure	15	18.52
HIV	42	51.85
Tuberculosis	29	35.80
Nephrotic syndrome	1	1.23
Recent admission	28	34.57
Recent surgery	11	13.58
Pregnancy	4	4.94
Obesity	5	6.15
Varicose vein	7	8.64
Immobility	9	11.11
Hormone replacement therapy	1	1.23
Oral contraceptive pill	4	4.92
Family history	2	2.47
Smoking	21	25.93

Analysis of haematological profile of the patients showed mean haemoglobin level of 9.76 g/dL (standard deviation 1.92) for female patients and median haemoglobin level of 13.1 g/dL (IQR 9.02 g/dL – 13.80 g/dL) for male patients. The median albumin level was 28.0 g/dL (IQR 23 g/dL – 36 g/dL). D-dimer was done in 31 (38.37%) patients, 30 of whom had elevated levels. Baseline International Normalised Ratio (INR) was done in 78/81 (93.71%) patients, of whom 51 (65.38%) had a range of 0–1.0, whilst INR on discharge was done in 69/81 (85.16%) patients. The discharge INR was in the therapeutic range (INR 2–3) for 23 patients (23/81, 28.4%), it was sub-therapeutic (INR < 2) for 39 patients (39/81, 48.1%), and for 7 (7/81, 8.61%) patients INR was > 3.

There was a 97.5% compliance with the South African Standard Treatment Guidelines (2012) for adults for whom Warfarin and Low Molecular Weight Heparin (LMWH) or low-dose unfractionated heparin (as an alternative where cost precludes the use of LMWH) were started simultaneously.^23^ However, only 34.18% had weight-based dosing of LMWH as majority of the patients had no documentary evidence of their weight. It was noted that the clinicians preferentially prescribed a convenient dosing of 80 mg twice daily of the LMWH (as majority of the patients [60.2%] were given this dose), possibly because of the non-documentary evidence of the weight. Warfarin was commenced at a low dose of 2.5 mg daily for 37 of the patients (37/81, 45.68%) and was titrated upward according to the baseline INR level to achieve a therapeutic range in only 23(of 81, 28.4%) of the patients.

## Discussion

The most common associated factors in the case series were HIV and TB. Although these findings differ from the risk factors identified in studies done in HIC, it is similar to the findings of a study conducted in GF Jooste Hospital in Cape Town, where HIV and TB were also identified as the most common risk factors accounting for 64.0% and 55.0%, respectively.^9,10,24^ That study showed that 97% of the DVT occurred in the lower limb, which is consistent with our finding of 96.3% of the DVT occurring in the lower extremity. A retrospective study conducted by Saber^25^ in Mount Sinai Hospital, New York, in 2001 showed that HIV infection increases the risk of DVT, especially in the lower extremity by 10-fold. A similar study by Matta,^26^ which analysed data from the National Hospital Discharge Survey from 1990 through 2005, also concluded that the incidence of VTE in patients with HIV infection was higher than in non-HIV patients. Considering this context-specific prevalence of HIV in South Africa and the documented strong association between HIV and thrombosis, it is not surprising that the risk of DVT would be increased in this population. There is also evidence to support the occurrence of DVT in patients with TB infection and this may be accounted for by the fact that TB is the most common opportunistic infection in HIV-infected people in Africa accounting for the synergistic effect causing an increased risk of developing DVT.^27,28,29^ In addition, many of these patients with HIV and TB coinfections may have had prolonged illnesses at home that may have contributed to their immobility, further predisposing them to DVT.

It is important to note that both Padau predictive rule and Wells criteria may need to be further modified for validation within South Africa to reflect the additional risk for DVT associated with these infections because they exclude HIV and TB, which were the two most common observed known risk factors in this study population. Further studies using appropriate designs and incorporating these scoring tools, which score HIV as risk factor, are recommended to test the validity of these tools for the local South African setting.

Despite existing evidence that age has a direct correlation with the formation of DVT resulting in an exponential increase in the incidence rate with increasing age, analysis of our study showed median age of 40 years (IOR 32–60 years).^30,31^ This finding is in keeping with studies done in similar setting with median age of 37 years (IQR 15–88 years).^24^ Similarly, the findings of Olubanwo^15^ showed that 68.6% of the DVT cases were found in those aged < 40 years. This could be explained by the fact that HIV is most prevalent in age group 15–49 years, which constituted the age group of the majority of our study sample (69.1%).^32^

Recent hospitalisation, smoking, previous DVT and congestive cardiac failure were the other risk factors observed in the study. However, risk factors such as malignancy, use of OCP, HRT and obesity were not common in the study and accounted for only 1.23% – 6.15%, which is consistent with the study of Levine et al.^33,34,35^ Recent hospital admissions occurred within the preceding 1 month in 28 patients (34.57%) of which the majority were because of HIV/AIDS and opportunistic coinfections. The results of studies investigating the relationship of smoking with VTE remain inconsistent and controversial. Whilst a prospective epidemiological study by Meyer-Michel Samama (the Sirius study)^36^ observed smoking as a protective factor for DVT, a systematic review and meta-analysis by Cheng et al.,^37^ which examined the link between smoking and VTE in the general population, found that smoking was associated with an absolute risk of 24.3 cases of VTE per 100 000 person-years. They concluded that smoking is associated with a slightly increased risk of VTE.^37^

The preponderance of female gender in our study population is in keeping with findings from earlier studies but inconsistent with the finding of Levine et al.^35^ of male predominance of 60%.^24,33,38^ The result showed that 29 patients (35.8%) developed DVT during admission for other medical conditions of which HIV accounted for the majority. This may have been prevented with appropriate pharmacologic thromboprophylaxis. The lack of recommendation for clear thrombosis treatment guideline for this growing sub-population (HIV-infected population) leaves the decision to the attending clinicians to either administer thromboprophylaxis or not, although guidelines advocate that any sick patient who is non-ambulant receive thromboprophylaxis, unless contraindicated (South African guideline).^39^ Further investigation using these suggested modified scoring tools to assess risk and identify potential high-risk hospitalised medical patients for appropriate thromboprophylaxis is required. Proactive management of these patients may reduce morbidity and mortality associated with DVT.

Baseline INR was done in 78/81 (93.71%) patients, of whom 51 (65.38%) had a range of 0–1.0, whilst INR on discharge was done in 69/81 (85.16%) patients. The discharge INR was in the therapeutic range (INR 2–3) for 23 patients (23/81, 28.4%); sub-therapeutic (INR < 2) for 39 patients (39/81, 48.1%) and INR was > 3 for 7 patients (7/81, 8.61%). The practical implications of optimising the INR in a busy district hospital in which staff are constantly pressured to make beds available because of the large burden of disease in South Africa sometimes contributes to the suboptimal care in that many patients are discharged before optimisation of the INR. Patients should ideally have INRs in the range of 2–3 before discharge but only a minority of patients were discharged with INRs in the therapeutic range. Outpatient management to obtain optimal INRs are done in first-world countries, but this is not feasible in the public sector in South Africa because of limited resources.^40,41^ The gap identified in the study will thus lend itself to a quality improvement project to improve the management of patients with DVT.

### Limitations of the study

Information bias existed in the study as it was not possible to standardise the quality of the patients’ clinical notes because of the retrospective nature of the study, therefore missed or improper documentation could not be completely ruled out. Furthermore, the study was done in an urban district hospital in a high prevalence of HIV and TB area, it is not possible to generalise the findings of the study.

### Recommendations

- Further review of the modified Wells criteria to include HIV with a possible score of 1 and validated for use in a population with high burden of HIV infection, for example, Sub-Saharan Africa.
- The authors propose that all sick, hospitalised HIV-infected patients should be considered for thromboprophylaxis, particularly if non-ambulant, and until such time as they are fully weight-bearing ambulant.
- It is important that practicing clinicians ensure that all hospitalised patients with VTE should be adequately anticoagulated prior to discharge and ideally followed up at an anticoagulation clinic.

## Conclusion

The review of the profile and risk factors of patients with DVT in our study setting shows predominantly young patients with female preponderance. TB and HIV are well-recognised risk factors, either alone or in conjunction. Clinicians should have a heightened awareness of VTE in patients with either condition or where both conditions occur together and appropriate thromboprophylaxis should be administered.
